# Analysis and validation of overall *N*-glycan conformation in *Privateer*

**DOI:** 10.1107/S2059798323003510

**Published:** 2023-05-23

**Authors:** Jordan S. Dialpuri, Haroldas Bagdonas, Mihaela Atanasova, Lucy C. Schofield, Maarten L. Hekkelman, Robbie P. Joosten, Jon Agirre

**Affiliations:** a York Structural Biology Laboratory, Department of Chemistry, University of York, York YO10 5DD, United Kingdom; b Oncode Institute and Division of Biochemistry, Netherlands Cancer Institute, Plesmanlaan 121, 1066 CX Amsterdam, The Netherlands; Diamond Light Source, United Kingdom

**Keywords:** glycobiology, validation, *Privateer*, *N*-glycans, torsion angles

## Abstract

The oligosaccharides in *N*-glycosylation provide key structural and functional contributions to a glycoprotein. These contributions are dependent on the composition and overall conformation of the glycans. The *Privateer* software allows structural biologists to evaluate and improve the atomic structures of carbohydrates, including *N*-glycans; this software has recently been extended to check glycan composition through the use of glycomics data. Here, a broadening of the scope of the software to analyse and validate the overall conformation of *N*-glycans is presented, focusing on a newly compiled set of glycosidic linkage torsional preferences harvested from a curated set of glycoprotein models.

## Abstract 2

The *Privateer* software allows structural biologists to evaluate and improve the atomic structures of carbohydrates, including *N*-glycans. This software has recently been extended to check glycan composition through the use of glycomics data, and the broadening of its scope is presented in this article.

## 1. Introduction

Post-translational modifications (PTMs) are covalent modifications of proteins that occur after the nascent polypeptide has left the ribosome. PTMs may induce significant changes in the structure and function of the protein (Xin & Radivojac, 2012 ▸). A fundamental and abundant PTM is *N*-glycosylation, in which an oligosaccharide moiety is attached to the N atom of an asparagine side chain in the target protein. The oligosaccharide is subsequently trimmed and modified according to the available cellular enzymes: glycoside hydrolases, glycosyl and oligosaccharyl transferases. The resulting oligosaccharide, or *N*-glycan, may end up having anything from a complex to a minimal composition, leading to a specific 3D conformation of the mature glycoprotein (Shental-Bechor & Levy, 2009 ▸). *N*-Glycosylation is key to all sorts of interactions, including those with cell-surface receptors (Petrescu *et al.*, 2006 ▸; Rudd *et al.*, 2004 ▸) or even other parts of the same glycoprotein, as shown in studies of the dynamics of SARS-CoV-2 spike, where conformational changes in the Asn165 glycan push up the receptor-binding domain of the spike (Casalino *et al.*, 2020 ▸).

Understanding the complex structure of carbohydrates is challenging due to the various stereochemical and regiochemical possibilities exhibited by *N*-glycans. Producing a correct 3D structure of a glycoprotein at a good enough resolution can be vital in understanding how some biological processes unfold. Alas, working with glycans in software for X-ray crystallography and electron cryo-microscopy has historically been all but straightforward: many carbohydrate modelling, refinement and validation processes relied on software written primarily for proteins and nucleic acids (Atanasova *et al.*, 2020 ▸), and libraries of restraints had become outdated or were incorrect (Agirre, 2017 ▸). While recent efforts have aimed to address this situation (Atanasova *et al.*, 2022 ▸; Joosten *et al.*, 2022 ▸), carbohydrate methodology still trails that designed for proteins.

Obtaining a glycoprotein structure at a high enough resolution can generally be considered to be more difficult than with a glycan-free protein. Two main issues are routinely identified as problematic when it comes to obtaining higher resolutions: heterogeneity and mobility, both of which translate into poorer experimental data. Owing to these complications, the Protein Data Bank (PDB; Berman *et al.*, 2000 ▸) contains models that include incorrect nomenclature (Lütteke *et al.*, 2005 ▸), impossible linkages (Crispin *et al.*, 2007 ▸) and improbably high-energy conformations of carbohydrates that deviate from the low-energy chair conformation of six-membered rings (Agirre, Davies *et al.*, 2015 ▸): in general, a ^4^ *C*_1_ chair for d-pyranosides and a ^1^ *C*_4_ chair for l-pyranosides. Ring conformations (Cremer & Pople, 1975 ▸) and their energetics (Davies *et al.*, 2012 ▸) are discussed in detail elsewhere. Using models with incorrect glycochemistry in downstream analyses or molecular simulations will cause misrepresentation and misinterpretation, while also perpetuating these errors. Software packages such as *pdb-care* and *CARP* (Lütteke *et al.*, 2005 ▸), and more recently *Privateer* (Agirre, Iglesias-Fernández *et al.*, 2015 ▸; Bagdonas *et al.*, 2020 ▸), can be utilized for the identification and rectification of these model errors, therefore allowing future refinement data libraries to be as accurate and representative as possible.

In this study, torsion angles (dihedral angles) in curated structures of *N*-glycan-forming pyranosides were collected in order to create accurate torsional libraries for use in the *Privateer* validation software. Previous torsional databases such as GlyTorsionDB (Lütteke *et al.*, 2005 ▸) and its associated link-checking tool (*CARP*) incorporate potentially flawed models from the PDB, as they pre-dated the introduction of ring conformation into the routine validation of glycan structures (Agirre, Iglesias-Fernández *et al.*, 2015 ▸); therefore, a survey of the PDB was completed with each PDB entry being analysed and validated using *Privateer* to ensure that the *N*-glycans were well fitted to the electron density without any conformational errors. Also, in order to avoid the presentation of data on multiple torsional plots and to allow the easy identification of standout (outlier) linkage conformations, a *Z*-score is calculated for each linkage, with standout linkages being highlighted in orange on glycan diagrams that follow the third edition of the Standard Symbol Nomenclature for Glycans (SNFG; Varki *et al.*, 2015 ▸). Furthermore, in recognition that not every standout linkage conformation will be the consequence of a modelling mistake, a collection of verified cases where the interaction between glycan and protein residues has caused an unusual conformation is presented. Finally, a similar study was completed using *PDB-REDO* (van Beusekom, Touw *et al.*, 2018 ▸) to analyse whether modern refinement techniques can lead to less frequent errors in the *N*-glycan models.

## 2. Materials and methods

### 2.1. Data-set collection and validation

A local PDB mirror (August 2021) was created for this study. The PDB mirror was then scanned for proteins containing glycosylated amino-acid residues. Of the monosaccharides contained within these chains, the conformations of the six-membered rings (pyranosides) were validated using *Privateer*: the software calculates ring conformation using the Cremer–Pople algorithm (Cremer & Pople, 1975 ▸) and then compares the detected ring conformation with the minimal energy conformation stored in an internal database. The data set was filtered to include only monosaccharides with a real-space correlation coefficient (RSCC) higher than 0.80 [RSCC (equation 1) is a measure of the local agreement between a portion of an atomic model and the observed electron-density map that surrounds it] and which had been deemed diagnostically correct by *Privateer*, *i.e.* no nomenclature errors, no unphysical puckering amplitude and all pyranosides in their minimal energy conformations (a chair in all analysed cases). *Privateer* checks that the anomeric and absolute stereochemistry in the structure matches that encoded in the three-letter code (for example that a monosaccharide modelled as MAN is perceived to be α-d-mannose), that the ring conformation matches the lowest energy pucker, which is a ^4^ *C*_1_ chair for most d-pyranosides, with special cases such as ^1^ *C*_4_ for the mannose moiety in tryptophan mannosylation (Akkermans *et al.*, 2022 ▸; Frank *et al.*, 2020 ▸), including puckering amplitude (Cremer & Pople, 1975 ▸).

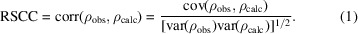

No resolution cutoffs were explicitly applied, although some filtering is implicit in requiring a minimum RSCC, as the accumulation of model-error components at low resolutions makes it harder to obtain high RSCC values. A total of 68 541 monosaccharides were analysed, 57 569 of which *Privateer* deemed correct; only these were used in the study. A further 8511 showed a high-energy ring conformation, which normally requires manual assessment. A total of 2421 monosaccharides showed geometry and/or nomenclature errors.

For the *PDB-REDO* comparison, the equivalent monosaccharides were taken from the so-called ‘conservatively optimized’ models in the PDB-REDO databank (van Beusekom, Touw *et al.*, 2018 ▸), *i.e.* models that were re-refined without any torsional restraints for carbohydrates but were not subjected to *N*-glycan rebuilding procedures (van Beusekom *et al.*, 2019 ▸).

**Figure 1:**
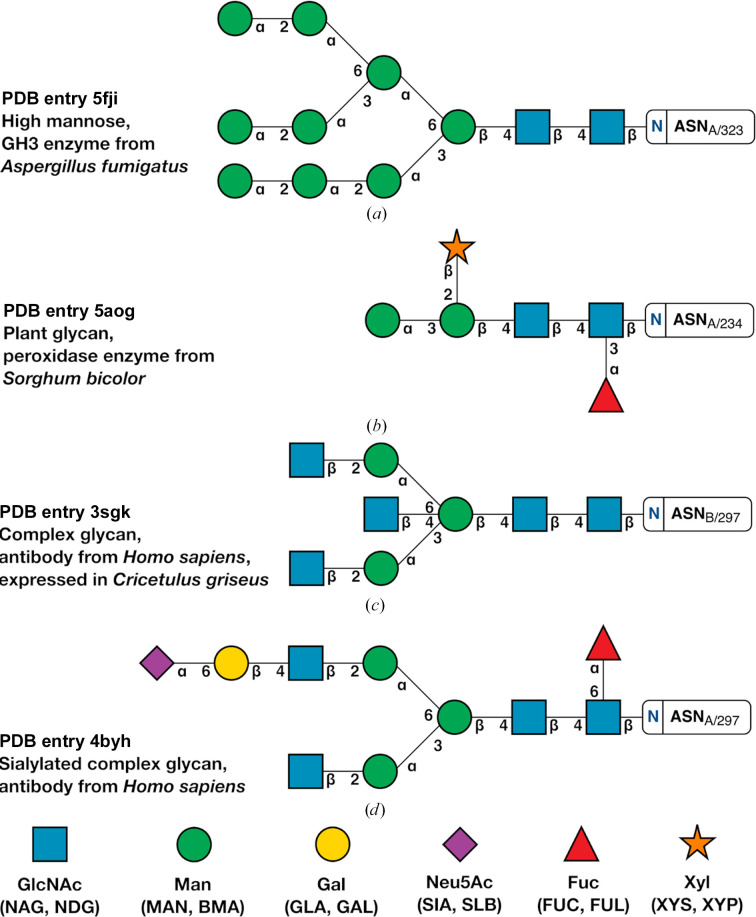
Examples of different types of *N*-glycans shown using the Symbol Nomenclature for Glycans (SNFG). The Greek letters and numbers show the *N*-glycan linkage naming. (*a*) High mannose from PDB entry 5fji, a GH3 glucosidase from *Aspergillus fumigatus* (Agirre *et al.*, 2016 ▸). (*b*) Plant glycan from PDB entry 5aog, a sorghum peroxidase (Nnamchi *et al.*, 2016 ▸). (*c*) PDB entry 3sgk (Ferrara *et al.*, 2011 ▸) shows a complex glycan from an Fc fragment of a human antibody, which was in turn expressed in CHO cells. (*d*) A sialylated complex glycan from PDB entry 4byh (Crispin *et al.*, 2013 ▸) expressed in *Homo sapiens*. This figure was produced with *Privateer*, which follows SNFG version 3 (Varki *et al.*, 2015 ▸).

**Figure 2:**
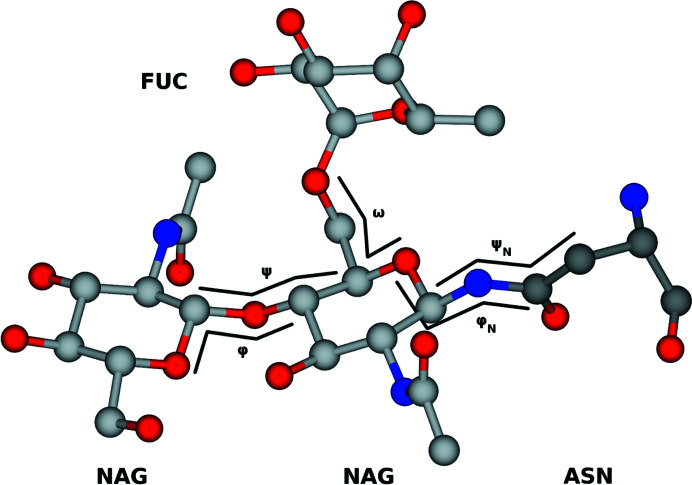
Visual representation of φ and ψ in both sugar–sugar linkages and the NAG-ASN linkage. This figure was generated from PDB entry 4byh (Crispin *et al.*, 2013 ▸).

Example linkages present in diverse glycans are shown in Fig. 1 ▸ using the third edition of the SNFG (Varki *et al.*, 2015 ▸), which *Privateer* implements. The definition of φ and ψ for *N*-acetyl-β-d-glucosamine (GlcNAc, or NAG in the PDB Chemical Component Dictionary) linked to asparagine, plus all 1–2, 1–3 and 1–4 glycosidic bonds, and additionally ω, which covers 1–6 bonds such as in α-d-mannose–1,6–α-d-mannose and α-l-fucose–1,6–*N*-acetyl β-d-glucosamine, is shown in Fig. 2 ▸. While completing this study, a large array of different linkages were identified; however, only a small number had enough independent observations to enable meaningful data extraction. Indeed, only approximately 10% of protein models deposited in the PDB contain one or more carbohydrate groups, while around 6% are *N*-glycosylated (Agirre, 2017 ▸). We set the minimum number of required observations to 50 and introduced a mechanism for *Privateer* to report which linkages could not be validated due to insufficient data (see below). A table of the linkages investigated in this study is given as Table 1 ▸, as well as the commonly used abbreviations associated with them.

### 2.2. Implementation in *Privateer*

To assess the normality of the torsion angles between monosaccharides in *N*-glycans, a *Z*-score system was implemented using similar methods to the *Tortoize* (van Beusekom, Joosten *et al.*, 2018 ▸) and *WHAT_CHECK* (Hooft *et al.*, 1997 ▸) software. The *Z*-score is based on how common a certain (φ, ψ) combination is compared with a reference set of the same glycosidic linkages calculated from high-quality structure models. To calculate the *Z*-scores, torsional data from each linkage were split into two-dimensional bins with a 2° bin spacing and formed into a database. The *Z*-score is calculated as described by Hooft *et al.* (1997 ▸) and shown in equation (2).

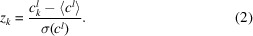

Let *k* be a particular glycosidic linkage, for example BMA402–NAG401 in a PDB file, under scrutiny and *z_k_* be its *Z*-score for the φ/ψ torsion pair measured on the structure; *l* is the linkage type (Man–β1,4–GlcNAc in this case), 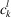 is the number of data points of that linkage (where *c* is a count) in the 2° × 2° bin corresponding to the φ/ψ torsion pair in the database, 〈*c_l_* 〉 is the average number of data points for that linkage across all bins and σ〈*c_l_* 〉 is the corresponding standard deviation of the number of data points for linkage *l* in the database, again across all bins. As derived from the formula, positive *Z*-scores indicate that the φ/ψ torsion pair is well represented in the database and thus normal, whereas negative *Z*-scores indicate the opposite. Also, the scores are normalized to make the results comparable between different linkages. Detailed results and their interpretation are discussed in the next section.

After scoring every glycosidic linkage, a global *Z*-score may be calculated by simply averaging the *Z*-scores of all *N*-glycan linkages. In addition to this, comparison to a reference set of PDB entries with *N*-glycans allowed the calculation of a relative ‘quality *Z*-score’, which is an additional parameter that can be used as a measure of glycan normality. The reference set was chosen following a set of criteria: crystallographic structures and reflections from the wwPDB with *R*_free_ < 0.25 and reported resolution ≤ 2.50 Å, with glycans longer than four pyranosides and with a composition backed up by a GlyConnect ID (Alocci *et al.*, 2019 ▸). As a result, 510 structures were chosen containing 59 unique glycan structures. The resolution range covered by the data set was 1.12–2.50 Å, and the *R*_work_ and *R*_free_ values were in the ranges 0.10–0.23 and 0.12–0.25, respectively.

To provide a visual means of highlighting those linkages with an unusual *Z*-score, the SNFG (Varki *et al.*, 2015 ▸) vector engine within *Privateer* (McNicholas & Agirre, 2017 ▸) was modified to create an orange background behind the linkages. Linkages for which insufficient data could be collected for validation are marked with a grey background. This representation was used in the figures presented in this study. The representation was also extended to cover the monosaccharides in glycans, so that interesting or problematic models can quickly be identified. We note that an orange background does not automatically mean that there is a modelling mistake, but rather that the linkage is worth inspecting.

## 3. Results and discussion

The number of *N*-glycosylated structures in the PDB is growing steadily (Scherbinina & Toukach, 2020 ▸; Agirre, 2017 ▸), supported by the introduction of carbohydrate structure modelling and validation tools such as *pdb-care* (Lütteke & von der Lieth, 2004 ▸), the *N*-glycan building module in *Coot* (Emsley & Crispin, 2018 ▸) and *Privateer* (Agirre, Iglesias-Fernández *et al.*, 2015 ▸). However, as the resolvability of pyranosides in *N*-glycans decreases the further the monosaccharides are from the asparagine residue (Atanasova *et al.*, 2020 ▸), the abundance of the data collected here dwindles for linkages that form the antennae of the glycans. As stated previously, we set a cutoff of 50 data points in order to guarantee the reliability of the *Z*-score calculation, and this necessarily means that some glycosidic linkages are not yet included in the analysis performed by the *Privateer* software. Scripts for reproducing and extending this work are included in the relevant section here, meaning that the torsion library can be regenerated in future when more data are available.

**Figure 3:**
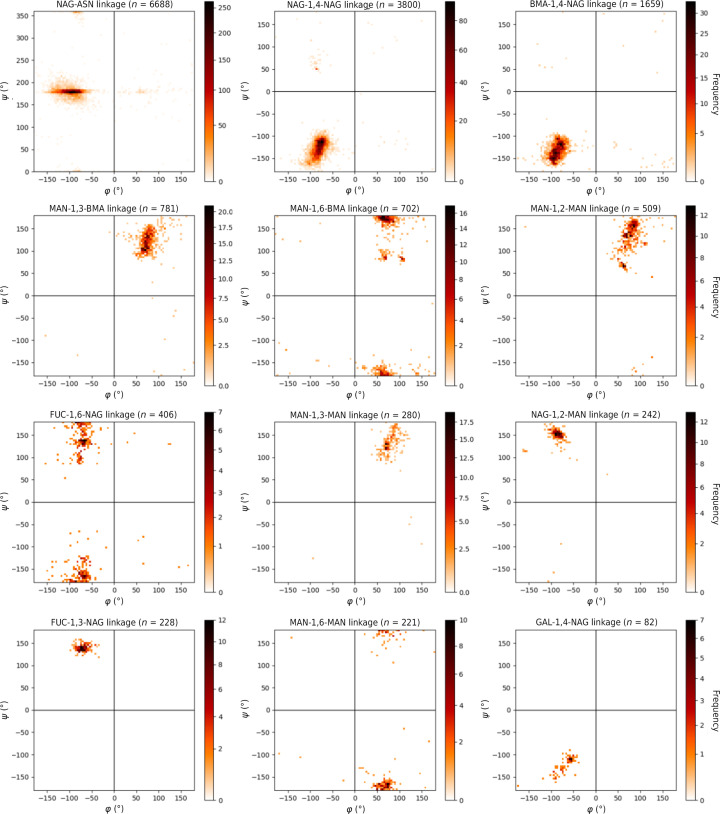
Plots of φ and ψ values for all linkages collected with over 50 data points. Colour bars are plotted using the power-law distribution (Clauset *et al.*, 2009 ▸) to highlight outliers visually. Plots allow visualization of the energy-minima values.

The torsional data that we harvested are plotted in Fig. 3 ▸. A first close inspection of the graphs reveals a straightforward correspondence between the most frequent linkage conformations for every link type and their calculated energy minimum or minima in the Disac3-DB section of the Glyco3D 2.0 database (Pérez *et al.*, 2015 ▸) and GlycoMapsDB (Frank *et al.*, 2007 ▸). The mean linkage torsion angles and respective standard deviations of this PDB survey are shown in Supplementary Tables S1 and S2. Supplementary Table S1 shows the values implemented into *Privateer*. A comparative plot of quality *Z*-scores for the curated data set versus the rest of the PDB is available in Supplementary Fig. S1. Low-quality *Z*-scores (*Z* < −2) may indicate serious problems with the overall quality of glycans in the structure model. High-quality *Z*-scores (*Z* > 2), particularly in low-resolution structure models, may indicate over-restraining of torsions in model refinement and may warrant further inspection, as previously shown for proteins (Sobolev *et al.*, 2020 ▸).

### 3.1. GlcNAc–asparagine bond

Investigations of the torsion-angle data set between the asparagine (ASN) amino-acid side chain and GlcNAc (NAG) highlight a perhaps unsurprising trend. The φ torsion-angle data set has a greater standard deviation (σ = 25.3°) when compared with the ψ torsion angle (σ = 22.1°). This is most likely due to the ψ torsion angle referring to a C—N bond which has a bond order of greater than one, analogous to a peptide bond. Indeed, the mean value of ψ is 178.5°, which is very similar to the 180° torsion angle expected for a peptide bond. Such a bond has limited torsional freedom. The φ torsion angle refers to a single bond which has more rotational freedom, leading to the increased spread of torsional data for φ.

**Figure 4:**
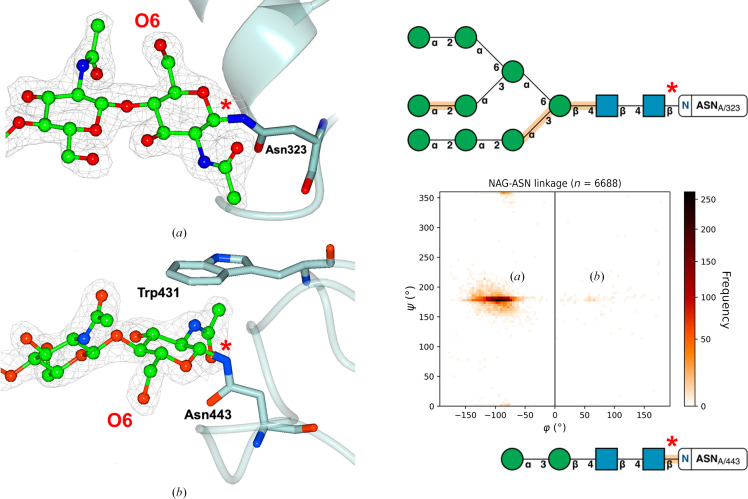
Two main conformations for the NAG-ASN bond are detected in our data set, as previously shown in the literature (Imberty & Perez, 1995 ▸). (*a*) shows the most frequent conformation (see the graph on the right for an idea of the numbers), with (*b*) showing a secondary and much more infrequent preference. In (*b*) the GlcNAc appears flipped with respect to the orientation it has in (*a*); this can be spotted easily by looking at O6 of GlcNAc (annotated in the figure), which appears on opposite sides of the asparagine side chain. Both shown conformations are from PDB entry 5fji (Agirre *et al.*, 2016 ▸); 2*mF*_o_ − *DF*_c_ electron density is shown at 1σ for the glycans, but is omitted for the asparagine side chains for reasons of clarity; the positions of the asparagine side chains showed a good fit to the electron density.

Correct modelling of the protein–sugar linkage torsion angle is particularly important to establish a good basis for other monosaccharides to be modelled further down the *N*-glycan tree. Two main conformations for NAG-ASN exist (Fig. 4 ▸), in which the conformation with a negative φ angle (Fig. 4 ▸ *a*) is the most abundant and the other conformation (Fig. 4 ▸ *b*), which is much more infrequent due to the additional CH–π interaction (Trp431) that is required to stabilize it, is flagged up as an outlier by *Privateer*. The arrangement shown in Fig. 4 ▸(*b*), found in a fungal GH3 β-glucosidase, is conserved across homologous structures.

### 3.2. Glycosidic linkages between pyranosides

*N*-Glycans exhibit common structures, as shown in Fig. 1 ▸. The similarity of these conformations explains the consistency in the types of linkages seen in various glycoproteins and allows this quantitative study. *N*-Glycosylated chains attach to the residue with a NAG sugar through a β-linkage. Attached to this initial NAG sugar through a β-1,4 linkage is an additional NAG sugar. This initial NAG-1,4-NAG linkage is abundant in the PDB and hence contains a large number (*n* = 3800) of validated data points. As evident by the two-dimensional histogram (Fig. 3 ▸), most NAG-1,4-NAG linkages contain torsion angles around φ ≃ −80° and ψ ≃ −130°.

Often, a BMA sugar is attached to the second NAG sugar through a β-1,4 linkage. This BMA-1,4-NAG linkage may theoretically have slightly more conformational variability than NAG-1,4-NAG due to its position further down the glycan tree; however, the spread of data (standard deviation) is similar for both NAG-1,4-NAG and BMA-1,4-NAG. In addition to this, in the complex tree a FUC sugar can be attached to the initial NAG through an α-1,6 linkage. The FUC-1,6-NAG linkage exhibits a large standard deviation around both torsion angles, particularly around the ψ angle. This could partially be the result of FUC being a terminal residue at this position in the glycan, but the FUC-1,3-NAG linkage, in which the FUC is also a terminal residue connected to the same NAG, has less spread in the observed torsion angles. A key difference, however, is the presence of a third torsion angle, ω, that gives more flexibility to the FUC-1,6-NAG linkage. This additional flexibility also leads to less well defined experimental data and thus more room for modelling errors.

Attachment of additional mannose sugars onto the *N*-glycan chain can often increase the amount of branching and the size of the chain (see Fig. 1 ▸ *a*). The most common attachment onto the currently terminal BMA sugar is MAN-1,3-BMA; indeed, this is shown in our data set of validated glycans (*n* = 781), with the positional isomer MAN-1,6-BMA being almost as frequent (*n* = 702). Interestingly, the MAN-1,3-BMA linkage exhibits standard deviations (ψ: σ = 22.6°) which are similar to those of NAG-1,4-NAG (ψ: σ = 22.8°). However, the MAN-1,6-BMA linkage torsion angles do not exist in a singular cluster and hence exhibit a larger standard deviation (ψ: σ = 33.3°). Again, this additional spread may be caused by the presence of a third torsion angle in the linkage.

Certain glycoproteins have further monosaccharide attachments such as a variety of MAN-MAN, NAG-MAN and SIA-GAL linkages. Interestingly, the torsion-angle spread for all MAN-MAN linkages (1,2, 1,3 and 1,6) is far greater than the torsion-angle spread for NAG-MAN torsion-angle data, despite having a similar data-set size and existing in a similar area of the protein. A reason for this may be the *N*-acetyl group in NAG, which makes the placement of the monomer in relatively poor density less error-prone. The large standard deviation of MAN-MAN linkages causes similar challenges to MAN-BMA linkages in torsional restraint application. As well as this, no apparent cluster was observed for the SIA-GAL linkage, most likely due to the very low number of deposited and curated linkages available in the data set. The values that φ can adopt appear to be determined by the anomeric form involved in the glycosidic linkage: for d-pyranosides this means −180° < φ < 0° for β-anomers and 0° < φ < 180° for α-anomers. The inverse is true for l-pyranosides.

Using this large torsion-angle data set, an investigation of torsion-angle spread with glycan chain length and branching was conducted, although no meaningful trend was identified between glycan chain length and torsion-angle standard deviation. Despite this, this large data set can be incorporated into software packages such as *Privateer* to improve the accuracy of glycoprotein models.

### 3.3. *PDB-REDO* analysis

With the increasingly commonplace solution of protein complexes with high-resolution data, it is imperative that model-building software can depict the conformation and position of *N*-glycans accurately. Through the comparison of *N*-glycan torsion angles of proteins deposited in the PDB and the PDB-REDO databank, the applicability and necessity of modern refinement techniques can be assessed. Comparisons between torsion angles in *N*-glycans deposited in the PDB and the PDB-REDO databank highlight an interesting relationship between structure resolution and torsion-angle accuracy, as shown in Table 2 ▸.

The PDB-REDO models used in this study had no torsional restraints applied during refinement. Therefore, the torsion angles calculated by *PDB-REDO* are not influenced by the potentially flawed torsional restraints applied before the model was initially deposited in the PDB. This application of consistent refinement techniques without torsional restraints leads to a data set which naturally has a larger spread than the PDB. To assess whether the PDB and PDB-REDO data sets are significantly different, a series of *t*-tests were performed and are summarized in Table 3 ▸.

For the NAG-1,4-NAG and BMA-1,4-NAG linkages, both mean torsion angles were deemed to be significantly different (*p* < 0.05) in the PDB and PDB-REDO data sets by the *t*-test. For the MAN-1,6-BMA linkage, while the φ angle was deemed to be significantly different, the ψ angle was not significantly different. Interestingly, both data sets showed no significant difference between both torsion angles for MAN-1,6-MAN and MAN-1,3-MAN linkages. While the PDB-REDO models had many occurrences in which the torsion angles were not statistically similar to those in the PDB data set, the torsion angles in both data sets are within one standard deviation of each other for every linkage. While it is impossible to automatically determine whether the glycosidic linkages in a deposited structure were restrained to certain values, we know that *PDB-REDO* does not apply torsional restraints. Hence, in the absence of potential bias towards torsion restraint targets, it is likely that the PDB-REDO databank represents a more realistic distribution of *N*-glycan glycosidic torsion angles and could be used as an alternative data source for validation in *Privateer*. A future update of *Privateer* will allow users to analyse their structures against either the PDB or PDB-REDO torsional sets.

**Figure 5:**
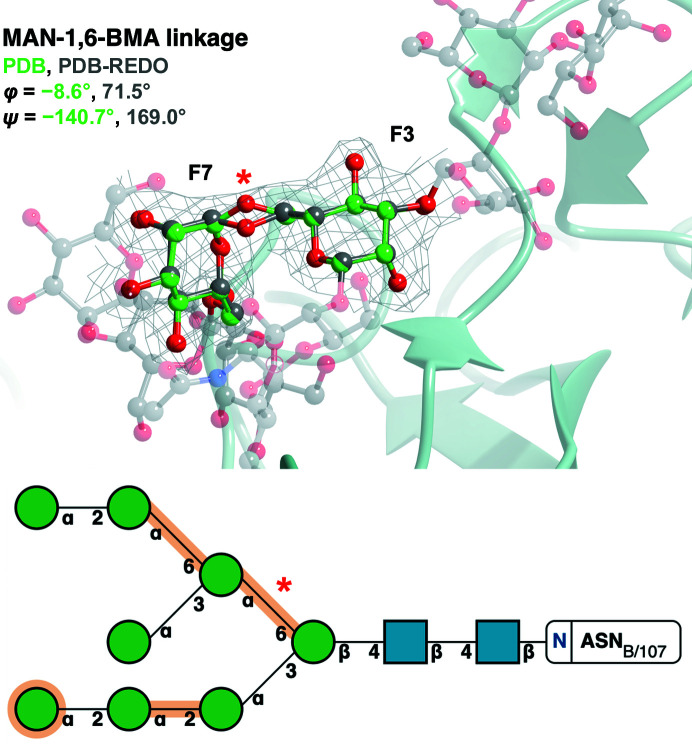
Refinement of PDB entry 6s2g (Ramirez-Escudero *et al.*, 2019 ▸) in *PDB-REDO* changes the torsion angle from an outlier in the PDB to an inlier in the PDB-REDO databank. The MAN (chain ID and sequence number F7)-1,6-BMA (chain ID and sequence number F3) linkage (red asterisk in the bottom panel) of PDB entry 6s2g (green) is identified as an outlier in the PDB (φ = −8.6°, ψ = −140.7°) but as an inlier in the PDB-REDO databank (φ = 71.5°, ψ = 169.0°): Δφ = 80.1°, Δψ = 50.3°. The change is brought on by moving the O6 atom (red asterisk in the top panel). BMA(F3) and MAN(F7) are represented by ball-and-stick models [C atoms in green (PDB model) or grey (PDB-REDO model)], whilst the rest of the attached glycan (PDB-REDO model) is represented in a faded grey ball-and-stick representation. 2*F*_o_ − *F*_c_ electron density (grey) is displayed for the linkage contoured to 1σ. The *Z*-scores for this linkage is −1.03 in the PDB model and 1.53 in the PDB-REDO model. The top image was produced using *CCP*4*mg*. Bottom: SNFG notation output from *Privateer*.

The application of consistent refinement techniques was also shown to improve outliers which had no physical basis for occurring (little clear interaction with residues or other ligands). Fig. 5 ▸ highlights the correction that *PDB-REDO* applies to the initially skewed MAN-1,6-BMA linkage. The data set of linkages originating from the PDB has numerous instances like this in which *PDB-REDO* corrects the torsion angles to more reasonable values. This powerful correction is another interesting and useful feature that *PDB-REDO* facilitates.

### 3.4. Outlier analysis

This analysis of *N*-glycan torsion angles deposited in the PDB reveals clusters of abundant torsion angles, as shown in Fig. 3 ▸. Perhaps due to the inherent variability in the environment surrounding monosaccharides in *N*-glycans, these torsion-angle clusters are spread over a large range in most cases. Outliers were quantified as any linkage which had a *Z*-score which was lower than −1. The *Z*-score reported here depends on the number of φ/ψ pairs relative to the database (Fig. 3 ▸) and not the deviation from the mean. The limit of −1 was chosen to highlight linkages that are uncommon in the database. Examining these linkages in further detail may highlight the cause of this. As always, surprising cases may either be chemically interesting to look at or be wrong. Here, we present one example of each.

#### 3.4.1. Electrostatic interactions

Repulsive and attractive electrostatic interactions are crucial for the functionality and stability of proteins (Law *et al.*, 2006 ▸). These interactions are facilitated by both positively charged (lysine and arginine) and negatively charged (glutamic acid and aspartic acid) amino-acid side chains. Similarly, these amino acids can affect the positions of monosaccharides contained within *N*-glycans via varying degrees of electrostatic interactions.

**Figure 6:**
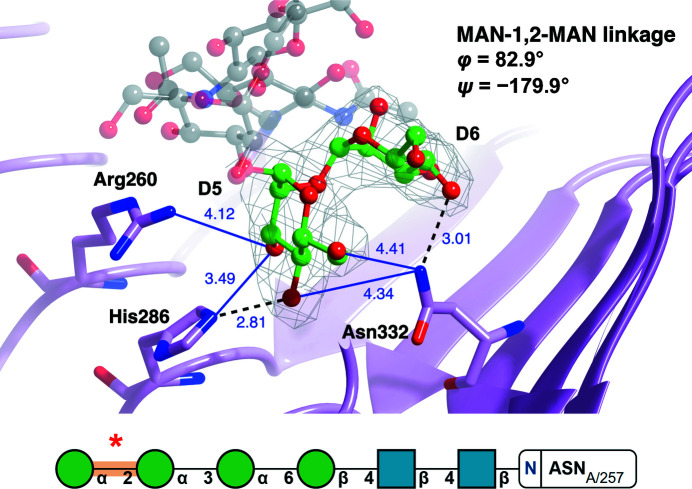
An unusual pair of MAN-1,2-MAN torsions in PDB entry 4j0m (She *et al.*, 2013 ▸). The mannose–mannose pair is well supported by the electron density, indicating that the unusual conformation of the linkage (red asterisk in the bottom panel) may be stabilized by interactions, electrostatic in this case, with surrounding side chains. The MAN (chain ID and sequence number D5)–MAN (chain ID and sequence number D6) linkage of PDB entry 4j0m (pink) is identified as an outlier (φ = 82.9°, ψ = −179.9°). The carbohydrate linkage is represented by a ball-and-stick model (C, green; O, red; N, blue). Residues identified as interacting with the linkage are represented by a cylindrical model (C, pink). Hydrogen bonds (black dashed line) and electrostatic interactions (within 4.5 Å, blue line) are shown with the distance between atoms in Å. 2*F*_o_ − *F*_c_ electron density (grey) is displayed for the linkage contoured to 1σ. Possible electrostatic interactions were identified for residues within 4.5 Å of the linkage and can be seen between Arg260 NH1 and MAN5 O3, His286 NE2 and MAN5 O3, Asn332 ND2 and MAN5 O4, and Asn332 ND2 and MAN5 O6. This linkage has a *Z*-score of −1.06. The top image was produced using *CCP*4*mg*. Bottom: SNFG notation output from *Privateer*.

Fig. 6 ▸ depicts an *N*-glycan (PDB entry 4j0m; She *et al.*, 2013 ▸) with MAN-1,2-MAN torsion angles that are highly deviated from the mean. Since this glycan has been validated using *Privateer* (all monosaccharides, including those involved in the linkage, were in low-energy chair conformations) and has an RSCC of greater than 0.80, indicating a good fit to electron density, it can be assumed that these torsion angles are a direct result of external factors. Upon examination of the area surrounding the glycan, it becomes evident that a network of electrostatic interactions could be affecting the conformation of the *N*-glycan chain. The proximity of the linkage to arginine, histidine and asparagine side chains may cause the observed deviation. Furthermore, this highlights how linkages further down a glycan tree can also be subject to interactions with protein residues. These interactions may also explain why MAN-MAN linkage torsion angles are less concentrated on one pair of values than the more constrained NAG-NAG linkage.

#### 3.4.2. High-energy ring-conformation anomalies may distort a linkage

**Figure 7:**
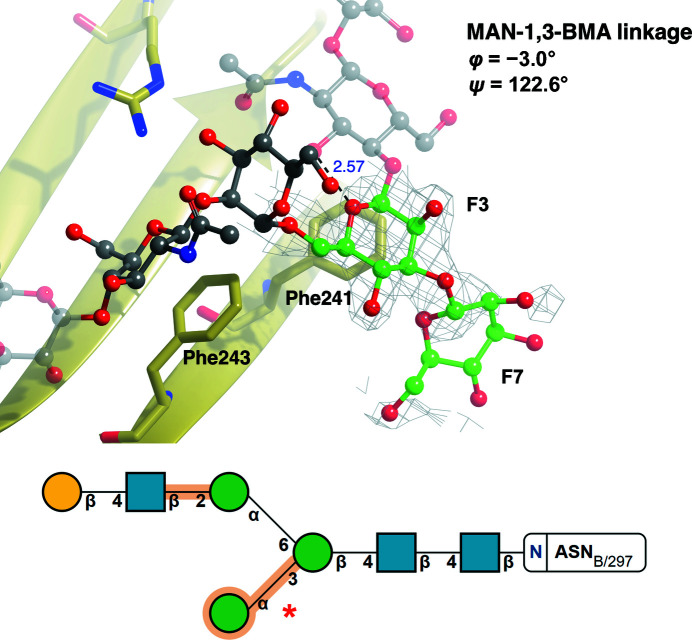
High-energy ring conformations may cause glycosidic link anomalies. The MAN(F7)–BMA(F3) linkage (red asterisk in the bottom panel) of PDB entry 5gsq (Chen *et al.*, 2017 ▸; gold), which was not part of the curated torsion-angle data set because the MAN residue has a poor RSCC, is identified as an outlier (φ = −3.0°, ψ = 122.6°). BMA (chain ID and sequence number F3) and MAN (chain ID and sequence number F7) are represented by a ball-and-stick model (C, green; O, red), whilst the rest of the attached glycan is shown in a faded grey ball-and-stick representation. Residues identified as interacting with the linkage are represented in stick form (C, gold; O, red; N, blue). Hydrogen bonds (black dashed lines) are shown with the distance between atoms in Å. 2*F*_o_ − *F*_c_ electron density (grey) is displayed for the linkage contoured to 1σ. Possible CH–π interactions were identified and can be seen between Phe243 and NAG(F5) and between Phe241 and BMA(F3). This linkage has a *Z*-score of −1.32, and presumably became distorted because the terminal mannose, MAN(F7), is in a ^1^ *S*_3_ skew-boat ring conformation (high energy; for further reading on conformational anomalies, please refer to Agirre, Davies *et al.*, 2015 ▸), as also highlighted in orange in the figure, due to the absence of well defined electron density. Both the linkage and ring conformations are unsupported by the electron density and should be either removed or corrected before deposition to reflect the most probable, low-energy conformations. The top image was produced using *CCP*4*mg*. Bottom: SNFG notation output from *Privateer*.

Fig. 7 ▸ shows a glycan stabilized by CH–π interactions with phenylalanine side chains (PDB entry 5gsq; Chen *et al.*, 2017 ▸). While the fit to electron density is reasonable for the first few pyranosides (which show no issues in the validation report), the MAN-1,3-BMA and the terminal MAN residue are highlighted in orange in the *Privateer* SNFG representation: the link has a *Z*-score of −1.32, indicating a large deviation, and the ring of the terminal mannose is in a ^1^ *S*_3_ conformation, which is wholly unexpected for a pyranoside that is part of an *N*-glycan and therefore is marked as worthy of inspection (orange). Examination of the electron-density map around the MAN-1,3-BMA pair reveals that the fit to the observed data is poor for the MAN residue; refinement against incomplete density usually results in high-energy ring conformations without the inclusion of torsion restraints (Agirre, 2017 ▸). The distortion of the ring conformation in pyranosides has been reported to have a knock-on effect on linkages (Agirre *et al.*, 2017 ▸); hence, we believe this is the most probable explanation for this outlier.

## 4. Conclusions

In this study, a large number and range of *N*-glycan linkage torsion angles were collected from both the PDB and the PDB-REDO databank after being curated using *Privateer*. The collected data, released and articulated through the *Privateer* software, will provide a strong foundation for future model building, refinement and validation software. The comparisons between the PDB and PDB-REDO models presented here assessed the importance of modern refinement techniques. The differences in the torsion angles between the validated PDB and PDB-REDO data sets are minimal. However, in certain cases the application of a consistent refinement technique can alleviate errors in the model-building process. Furthermore, the absence of torsional restraints in PDB-REDO perhaps allows a more realistic spread of torsional values to be observed. It is also important to note valid rationalizations for linkage torsion angles deviating from the calculated mean. Electrostatic and steric interactions play a large role in protein folding in general and can cause or stabilize the skewed *N*-glycan linkage torsions exhibited in certain glycoproteins. Therefore, it is highly likely that these electrostatically charged or sterically bulky amino acids play a role in overall *N*-glycan conformation.

## 5. Availability and open research data

All scripts, data and graphics associated with this work have been uploaded to Zenodo (https://doi.org/10.5281/zenodo.7356467). The *Privateer* source code is available from GitHub (https://github.com/glycojones/privateer). Binaries will be released as an update to *CCP*4 8.0.

## Supplementary Material

Supplementary Tables and Figure. DOI: 10.1107/S2059798323003510/qe5003sup1.pdf

Associated scripts, data and graphics: https://doi.org/10.5281/zenodo.7356467

Privateer source code: https://github.com/glycojones/privateer

## Figures and Tables

**Table 1:** Full names, linkage abbreviations and shorthand notations with PDB Chemical Component Dictionary (CCD) codes for those linkages with sufficient data No anomeric data are displayed for CCD codes, as this information is integrated into the codes themselves; for example MAN is α-D-mannose and BMA is β-D-mannose.

Full linkage denomination	Abbreviation	CCD code
*N*-Acetyl-β-D-glucosamine–asparagine	GlcNAc–β–Asn	NAG-ASN
*N*-Acetyl-β-D-glucosamine–1,4–*N*-acetyl-β-D-glucosamine	GlcNAc–β–GlcNAc	NAG-1,4-NAG
β-D-Mannose–1,4–*N*-acetyl-β-D-glucosamine	Man–β1,4–GlcNAc	BMA-1,4-NAG
α-D-Mannose–1,3–β-D-mannose	Man–α1,3–Man	MAN-1,3-BMA
α-D-Mannose–1,6–β-D-mannose	Man–α1,6–Man	MAN-1,6-BMA
α-D-Mannose–1,2–α-D-mannose	Man–α1,2–Man	MAN-1,2-MAN
α-D-Mannose–1,3–α-D-mannose	Man–α1,3–Man	MAN-1,3-MAN
α-D-Mannose–1,6–α-D-mannose	Man–α1,6–Man	MAN-1,6-MAN
α-L-Fucose–1,3–*N*-acetyl-β-D-glucosamine	Fuc–α1,3–GlcNAc	FUC-1,3-NAG
α-L-Fucose–1,6–*N*-acetyl-β-D-glucosamine	Fuc–α1,6–GlcNAc	FUC-1,6-NAG
*N*-Acetyl-β-d-glucosamine–1,2–α-D-mannose	GlcNAc–β1,2–Man	NAG-1,2-MAN
β-D-Galactose–1,4–*N*-acetyl-β-D-glucosamine	Gal–β1,4–GlcNAc	GAL-1,4-NAG
α-Sialic acid–2,6–β-D-galactose	Sia–α2,6–Gal	SIA-2,6-GAL

**Table 2:** Comparison between the PDB and PDB-REDO torsional data Values have been rounded to the nearest integer due to the large deviations that were encountered.

		φ (°)		ψ (°)		
Resolution (Å)	Linkage	PDB	PDB-REDO	PDB	PDB-REDO	No. of entries
*x* < 1.50	NAG-1,4-NAG	−79 ± 8	−79 ± 24	−127 ± 18	−126 ± 26	132
1.50 < *x* < 3.00	NAG-1,4-NAG	−80 ± 13	−74 ± 24	−127 ± 23	−125 ± 24	3190
*x* > 3.00	NAG-1,4-NAG	−83 ± 24	−67 ± 36	−130 ± 23	−135 ± 27	472
**All**	**NAG-1,4-NAG**	**−80 ± 14**	**−73 ± 26**	**−127 ± 23**	**−126 ± 25**	**3800**
						
*x* < 1.50	BMA-1,4-NAG	−82 ± 10	−84 ± 10	−125 ± 14	−122 ± 13	37
1.50 < *x* < 3.00	BMA-1,4-NAG	−87 ± 16	−79 ± 29	−133 ± 18	−136 ± 23	1369
*x* > 3.00	BMA-1,4-NAG	−85 ± 26	−65 ± 47	−134 ± 21	−142 ± 26	250
**All**	**BMA-1,4-NAG**	**−87 ± 18**	**−77 ± 32**	**−133 ± 18**	**−137 ± 24**	**1659**
						
*x* < 1.50	MAN-1,6-BMA	69 ± 6	70 ± 5	150 ± 45	149 ± 45	17
1.50 < *x* < 3.00	MAN-1,6-BMA	72 ± 24	67 ± 24	167 ± 33	167 ± 34	606
*x* > 3.00	MAN-1,6-BMA	79 ± 42	66 ± 41	177 ± 31	179 ± 34	75
**All**	**MAN-1,6-BMA**	**72 ± 27**	**66 ± 26**	**168 ± 33**	**168 ± 35**	**702**
						
*x* < 1.50	MAN-1,3-BMA	77 ± 14	76 ± 14	122 ± 21	122 ± 21	23
1.50 < *x* < 3.00	MAN-1,3-BMA	75 ± 16	69 ± 20	121 ± 21	126 ± 23	602
*x* > 3.00	MAN-1,3-BMA	82 ± 21	68 ± 26	125 ± 30	135 ± 34	130
**All**	**MAN-1,3-BMA**	**76 ± 17**	**69 ± 21**	**121 ± 23**	**127 ± 26**	**777**
						
*x* < 1.50	MAN-1,6-MAN	60 ± 6	60 ± 3	−179 ± 6	−177 ± 4	8
1.50 < *x* < 3.00	MAN-1,6-MAN	67 ± 19	65 ± 20	−173 ± 16	−171 ± 16	175
*x* > 3.00	MAN-1,6-MAN	83 ± 45	68 ± 46	−174 ± 34	−180 ± 49	38
**All**	**MAN-1,6-MAN**	**68 ± 25**	**65 ± 25**	**−173 ± 20**	**−173 ± 24**	**221**
						
*x* < 1.50	MAN-1,2-MAN	73 ± 12	72 ± 12	126 ± 37	125 ± 37	23
1.50 < *x* < 3.00	MAN-1,2-MAN	77 ± 16	70 ± 16	134 ± 33	139 ± 35	387
*x* > 3.00	MAN-1,2-MAN	82 ± 25	71 ± 28	125 ± 26	130 ± 30	94
**All**	**MAN-1,2-MAN**	**78 ± 18**	**71 ± 19**	**132 ± 32**	**137 ± 35**	**507**
						
*x* < 1.50	MAN-1,3-MAN	74 ± 5	73 ± 6	118 ± 17	118 ± 18	9
1.50 < *x* < 3.00	MAN-1,3-MAN	77 ± 16	75 ± 16	133 ± 22	135 ± 24	234
*x* > 3.00	MAN-1,3-MAN	89 ± 18	83 ± 19	129 ± 34	130 ± 33	36
**All**	**MAN-1,3-MAN**	**78 ± 17**	**76 ± 16**	**132 ± 24**	**134 ± 25**	**280**

**Table 3:** Results of *t*-tests between the PDB and PDB-REDO data sets at all resolutions Values that are not significantly different (*p* > 0.05) are shown in bold.

Linkage	Resolution range (Å)	*t*-test result: φ	*t*-test result: ψ
NAG-1,4-NAG	0.93–6.92	Significantly different (*p* ≤ 0.05)	Significantly different (*p* ≤ 0.05)
BMA-1,4-NAG	1.20–8.69	Significantly different (*p* ≤ 0.05)	Significantly different (*p* ≤ 0.05)
MAN-1,6-BMA	1.20–6.92	Significantly different (*p* ≤ 0.05)	**Not significantly different (*p* = 0.34)**
MAN-1,3-BMA	1.20–6.92	Significantly different (*p* ≤ 0.05)	**Not significantly different (*p* = 0.39)**
MAN-1,6-MAN	1.12–6.31	**Not significantly different (*p* = 0.14)**	**Not significantly different (*p* = 0.35)**
MAN-1,2-MAN	1.20–6.92	Significantly different (*p* ≤ 0.05)	**Not significantly different (*p* = 0.18)**
MAN-1,3-MAN	1.20–6.31	**Not significantly different (*p* = 0.12)**	**Not significantly different (*p* = 0.56)**
