# Supplementary material for: Analysis and validation of overall N-glycan conformation in Privateer
Source: Acta Crystallogr D Struct Biol. 2023 May 23;79(Pt 6):462–72. doi: 10.1107/S2059798323003510 (PMC10233620; doi:10.1107/S2059798323003510)
Supplement: Supplementary file 1 [file d-79-00462-sup1.pdf]

STRUCTURAL BIOLOGY

Volume 79 (2023)

Supporting information for article:

Analysis and validation of overall N-glycan conformation in *Privateer*

Jordan S. Dialpuri, Haroldas Bagdonas, Mihaela Atanasova, Lucy C. Schofield, Maarten L. Hekkelman, Robbie P. Joosten and Jon Agirre

Table S1 Raw data from the survey for linkages containing more than 50 data points.

The number of data points in each data set is shown as well as the mean and standard deviation for each torsion angle value.

**Linkage**	**Number in data set**	**φ (°)**	***σ* φ (°)**	**ψ (°)**	***σ* ψ (°)**
ASN-NAG	6688	-97	25	179	22
NAG-1,4-NAG	3800	-80	14	-127	23
BMA-1,4-NAG	1659	-87	18	-133	18
MAN-1,3-BMA	781	76	18	121	23
MAN-1,6-BMA	702	72	27	168	33
MAN-1,2-MAN	509	78	18	132	32
MAN-1,3-MAN	280	78	17	132	24
MAN-1,6-MAN	221	68	25	-173	20
FUC-1,6-NAG	406	-74	24	167	41
FUC-1,3-NAG	228	-70	10	139	7
NAG-1,2-MAN	242	-86	15	151	15
GAL-1,4-NAG	84	-67	22	-122	24

Table S2 Raw data from the survey for 1,6 linkages that have an ω torsion angle.

Linkage	Number in data set	ω (°)	σω (°)
NAG-1,6-FUC	405	-58	43
BMA-1,6-MAN	701	18	70
MAN-1,6-MAN	221	-45	55

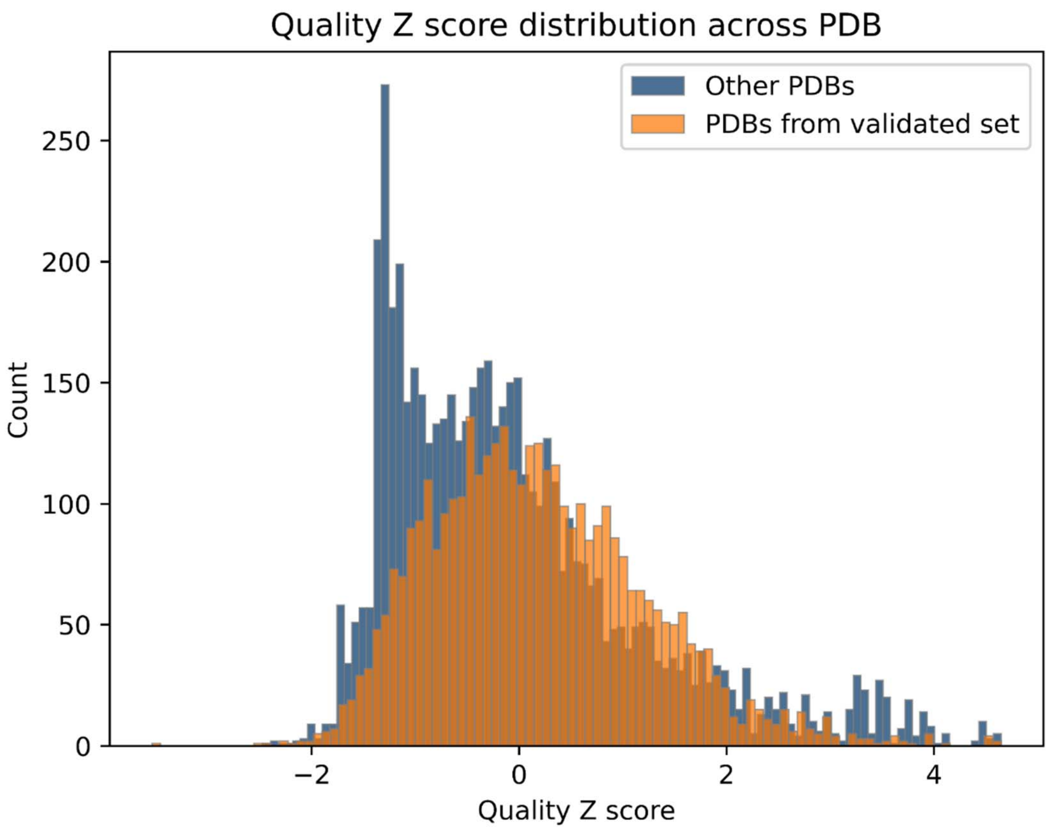

Figure S1 The distribution of quality Z scores across the PDB, the orange bars represent models which had at least one entry (glycan linkage) in our dataset of curated linkages. The blue bars represent the rest of the models in the PDB that contain *N*-glycans.
